# (10*E*,12*E*,14*E*)-9,16-Dioxoocta­deca-10,12,14-trienoic acid

**DOI:** 10.1107/S1600536812027870

**Published:** 2012-08-01

**Authors:** Lise Bréant, Catherine Vonthron-Sénécheau, Lydia Brelot, Annelise Lobstein

**Affiliations:** a Laboratoire de Pharmacognosie, UMR 7200, CNRS-Unistra, Faculté de Pharmacie, Université de Strasbourg, 74 route du Rhin, CS 60024, 67401 Illkirch Cedex, France; b Institut de Chimie de Strasbourg, UMR 7177 CNRS-Unistra, Service de Radiocristallographie, 1 rue Blaise Pascal, 67008 Strasbourg Cedex, France

## Abstract

The title octa­deca­trienoic acid derivative, C_18_H_26_O_4_, was isolated from *Silene maritima* With. (*Caryophyllaceae*), the first time this natural compound has been found in the *Caryophyllales* order. This fatty acid has an 18-carbon backbone with three double bonds on *trans* (*E*) conformation and two carbonyl. In the crystal, molecules are linked *via* pairs of O—H⋯O hydrogen bonds, forming inversion dimers.

## Related literature

For botanical information about *Silene maritima* With., see: Baker (1978 ▶); Bremer *et al.* (2009 ▶). For interactions between heavy-metals and *Silene maritima* With., see: Price & Abrahams (1994 ▶). For phytochemical investigation on *Silene maritima* With., see: Adrian-Romero *et al.* (1998 ▶). For previous descriptions of the title compound, see: Herz & Kulanthaivel (1984 ▶); Li *et al.* (2011 ▶). For lipoxygenase action on α-linoleic acid, see: Vellosillo *et al.* (2007 ▶). For environmental-stress-response involvement of oxylipines and their structure similarity with the title compound, see: Browse (2005 ▶); Schaller *et al.* (2004 ▶); Wasternack (2007 ▶). 
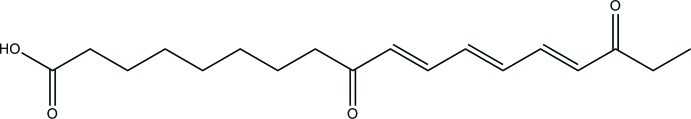


## Experimental

### 

#### Crystal data

C_18_H_26_O_4_
*M* *_r_* = 306.39
Triclinic, 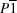

*a* = 5.6859 (3) Å
*b* = 7.7535 (5) Å
*c* = 19.9045 (16) Å
α = 81.333 (4)°
β = 84.152 (4)°
γ = 87.660 (4)°
*V* = 862.68 (10) Å^3^
*Z* = 2
Mo *K*α radiation
μ = 0.08 mm^−1^
*T* = 173 K
0.40 × 0.30 × 0.10 mm

#### Data collection

Nonius KappaCCD diffractometer
8217 measured reflections
3846 independent reflections
2648 reflections with *I* > 2σ(*I*)
*R*_int_ = 0.063

#### Refinement

*R*[*F* ^2^ > 2σ(*F* ^2^)] = 0.065
*wR*(*F* ^2^) = 0.189
*S* = 1.06
3846 reflections
204 parameters
H atoms treated by a mixture of independent and constrained refinement
Δρ_max_ = 0.27 e Å^−3^
Δρ_min_ = −0.24 e Å^−3^

### 

Data collection: *COLLECT* (Nonius, 1998 ▶); cell refinement: *DENZO* (Otwinowski & Minor, 1997 ▶); data reduction: *DENZO*; program(s) used to solve structure: *SHELXS97* (Sheldrick, 2008 ▶); program(s) used to refine structure: *SHELXL97* (Sheldrick, 2008 ▶); molecular graphics: *PLATON* (Spek, 2009 ▶); software used to prepare material for publication: *SHELXL97*.

## Supplementary Material

Crystal structure: contains datablock(s) I, global. DOI: 10.1107/S1600536812027870/zj2081sup1.cif

Structure factors: contains datablock(s) I. DOI: 10.1107/S1600536812027870/zj2081Isup2.hkl

Supplementary material file. DOI: 10.1107/S1600536812027870/zj2081Isup3.cml

Additional supplementary materials:  crystallographic information; 3D view; checkCIF report

## Figures and Tables

**Table 1:** Hydrogen-bond geometry (Å, °)

*D*—H⋯*A*	*D*—H	H⋯*A*	*D*⋯*A*	*D*—H⋯*A*
O2—H2⋯O1^i^	0.90 (4)	1.78 (4)	2.658 (2)	167.0 (4)

Symmetry code: (i) 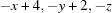.

**Fig. 1.**
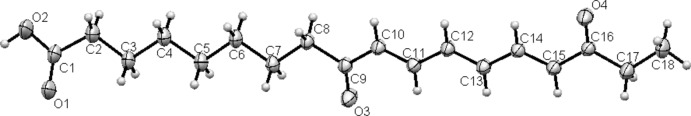
ORTEP representation of 9,16-dioxo-10E,12E,14E octadecatrienoic acid with 50% probability displacement ellipsoids for non-H atoms.

**Fig. 2.**
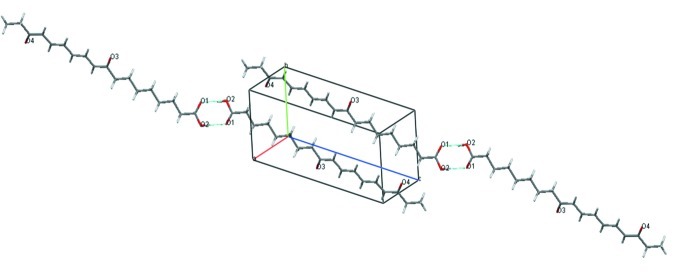
Packing diagram of four molecules of 9,16-dioxo-10E,12E,14E octadecatrienoic acid.

## supplementary crystallographic information

### Comment

*Silene maritima* With. belongs to the *Caryophyllaceae* family (Bremer *et al.*, 2009) and is a perennial species found on cliffs and shingle beaches in coastal habitats (Baker, 1978). This species is known to be a heavy-metal indicator (Price & Abrahams, 1994), and the only phytochemical investigation previously carried out on its aerial parts has revealed the presence of glycinebetaine, a compound used by cells for protection against osmotic stress (Adrian-Romero *et al.*, 1998).

This study is the first report of the presence of 9,16-dioxo-10E,12E,14E octadecatrienoic acid in the Caryophyllales order. This compound has previously been described only in the *Asteraceae* (Herz & Kulanthaivel, 1984) and *Lamiaceae* (Li *et al.*, 2011) families.

Its molecular structure contains an 18-carbon backbone with three double bonds in the *trans* conformation and two carbonyls (Fig. 1). The existence of intermolecular hydrogen interactions between two carboxylic functions was also observed (Fig. 2). The structure of this fatty acid might involve a lipoxygenase action on the α-linoleic acid (Vellosillo *et al.*, 2007). Thus suggesting that it could belong to oxylipines, a class of compounds implicated in environmental stress responses (Browse, 2005; Schaller *et al.*, 2004; Wasternack, 2007).

### Experimental

The sampling station is situated in the littoral zone of the western coast of Brittany (Brélès 29, France). Sampling was carried out in July 2008. The aerial parts of the plant were collected, air-dried, and grinded into a fine powder using a grinder (Retsch, ZM 200). Hydroalcoholic extract of aerial parts (1 kg) was prepared by soaking it at room temperature in 3 *x* 10 l of EtOH/H_2_O (6/4, *v/v*) during first 14 h, then 4 h and again 4 h, until exhaustion of raw materials. The extract was then filtered and dried under vacuum using a rotavapor. The amorphous solid, a black-brownish mass, was then dissolved in *d*-H_2_O and extracted sequentially with cyclohexane, CH_2_Cl_2_, AcOEt and *n-*BuOH. The CH_2_Cl_2_ extract (2.496 g) was fractionated on a silica gel column (SI60 0.050–0.16 mm in size, Merck) eluted successively with cyclohexane (500 ml), AcOEt (1170 ml) and MeOH (330 ml) to yield five main fractions. The third fraction (210 mg) was re-dissolved in MeOH and subjected to semi-preparative HPLC purification (Gilson, binary solvent system). The isolation was performed with a reverse phase Nucleodur C18 ec (250 mm *x* 21 mm, 5 µm) from Macherey-Nagel. Eluent A was H_2_O with 0.01% HCOOH, and eluent B was ACN. The flow rate was 10 ml/min and the injection volume was 400 µl at 40 mg ml^-1^. The elution conditions applied were: 0–5 min, linear gradient from 10% to 15% B; 5–55 min, 15% to 65% B; 55–60 min, 65% to 100% B; 60–70 min, 100% B isocratic. Simultaneous UV monitoring was set at 316 nm. This experimental procedure allowed us to isolate the title compound C_18_H_26_O_4_ at the retention time of 26 min. The pure compound (1 mg) was re-dissolved in 0.2 ml of MeOH/CHCl_3_ (2/1). The corresponding crystals of 9,16-dioxo-10E,12E,14E octadecatrienoic acid were grown thanks to a slow solubility decrease during two weeks at room temperature after addition of *n-*heptane (0.4 ml).

### Refinement

The H atoms, except for the H-atom of the carboxyl group which was located from Fourier difference maps, were positioned geometrically and refined using a riding model, with C—H = 0.95–0.99 Å and with *U*_iso_(H) = 1.2 (1.5 for methyl groups) times *U*_eq_(C).

### Crystal data

**Table:**

C_18_H_26_O_4_	*Z* = 2
*M**_r_* = 306.39	*F*(000) = 332
Triclinic, *P*1	*D*_x_ = 1.180 Mg m^−^^3^
Hall symbol: -P 1	Mo *K*α radiation, λ = 0.71073 Å
*a* = 5.6859 (3) Å	Cell parameters from 12739 reflections
*b* = 7.7535 (5) Å	θ = 1.0–27.5°
*c* = 19.9045 (16) Å	µ = 0.08 mm^−^^1^
α = 81.333 (4)°	*T* = 173 K
β = 84.152 (4)°	Plate, colorless
γ = 87.660 (4)°	0.40 × 0.30 × 0.10 mm
*V* = 862.68 (10) Å^3^	

### Data collection

**Table:**

Nonius KappaCCD diffractometer	2648 reflections with *I* > 2σ(*I*)
Radiation source: fine-focus sealed tube	*R*_int_ = 0.063
Graphite monochromator	θ_max_ = 27.5°, θ_min_ = 1.0°
phi and ω scans	*h* = −7→7
8217 measured reflections	*k* = −10→10
3846 independent reflections	*l* = −25→25

### Refinement

**Table:**

Refinement on *F*^2^	Primary atom site location: structure-invariant direct methods
Least-squares matrix: full	Secondary atom site location: difference Fourier map
*R*[*F*^2^ > 2σ(*F*^2^)] = 0.065	Hydrogen site location: inferred from neighbouring sites
*wR*(*F*^2^) = 0.189	H atoms treated by a mixture of independent and constrained refinement
*S* = 1.06	*w* = 1/[σ^2^(*F*_o_^2^) + (0.0932*P*)^2^ + 0.1679*P*] where *P* = (*F*_o_^2^ + 2*F*_c_^2^)/3
3846 reflections	(Δ/σ)_max_ < 0.001
204 parameters	Δρ_max_ = 0.27 e Å^−^^3^
0 restraints	Δρ_min_ = −0.24 e Å^−^^3^

### Special details

**Table:**

Geometry. All e.s.d.'s (except the e.s.d. in the dihedral angle between two l.s. planes) are estimated using the full covariance matrix. The cell e.s.d.'s are taken into account individually in the estimation of e.s.d.'s in distances, angles and torsion angles; correlations between e.s.d.'s in cell parameters are only used when they are defined by crystal symmetry. An approximate (isotropic) treatment of cell e.s.d.'s is used for estimating e.s.d.'s involving l.s. planes.
Refinement. Refinement of *F*^2^ against ALL reflections. The weighted *R*-factor *wR* and goodness of fit *S* are based on *F*^2^, conventional *R*-factors *R* are based on *F*, with *F* set to zero for negative *F*^2^. The threshold expression of *F*^2^ > σ(*F*^2^) is used only for calculating *R*-factors(gt) *etc*. and is not relevant to the choice of reflections for refinement. *R*-factors based on *F*^2^ are statistically about twice as large as those based on *F*, and *R*- factors based on ALL data will be even larger.

### Fractional atomic coordinates and isotropic or equivalent isotropic displacement parameters (Å^2^)

**Table:**

	*x*	*y*	*z*	*U*_iso_*/*U*_eq_	
C1	1.7412 (3)	0.9602 (2)	0.07113 (9)	0.0370 (4)	
C2	1.5281 (3)	0.9351 (3)	0.12198 (10)	0.0426 (5)	
H2A	1.3914	0.9132	0.0977	0.051*	
H2B	1.4935	1.0453	0.1410	0.051*	
C3	1.5485 (3)	0.7882 (2)	0.18074 (9)	0.0355 (4)	
H3A	1.6700	0.8163	0.2094	0.043*	
H3B	1.5999	0.6793	0.1625	0.043*	
C4	1.3134 (3)	0.7601 (2)	0.22433 (9)	0.0371 (4)	
H4A	1.2613	0.8705	0.2413	0.044*	
H4B	1.1935	0.7311	0.1954	0.044*	
C5	1.3238 (3)	0.6162 (2)	0.28491 (9)	0.0349 (4)	
H5A	1.4352	0.6489	0.3157	0.042*	
H5B	1.3859	0.5074	0.2684	0.042*	
C6	1.0834 (3)	0.5817 (2)	0.32511 (9)	0.0344 (4)	
H6A	1.0212	0.6904	0.3417	0.041*	
H6B	0.9720	0.5489	0.2944	0.041*	
C7	1.0945 (3)	0.4374 (2)	0.38581 (9)	0.0332 (4)	
H7A	1.2019	0.4722	0.4173	0.040*	
H7B	1.1622	0.3300	0.3693	0.040*	
C8	0.8539 (3)	0.3977 (2)	0.42492 (8)	0.0310 (4)	
H8A	0.7810	0.5074	0.4381	0.037*	
H8B	0.7506	0.3545	0.3942	0.037*	
C9	0.8631 (3)	0.2648 (2)	0.48833 (9)	0.0295 (4)	
C10	0.6343 (3)	0.2012 (2)	0.52376 (8)	0.0300 (4)	
H10	0.4917	0.2397	0.5045	0.036*	
C11	0.6245 (3)	0.0905 (2)	0.58252 (9)	0.0299 (4)	
H11	0.7696	0.0515	0.6002	0.036*	
C12	0.4081 (3)	0.0265 (2)	0.62080 (9)	0.0298 (4)	
H12	0.2623	0.0608	0.6027	0.036*	
C13	0.4038 (3)	−0.0797 (2)	0.68114 (9)	0.0303 (4)	
H13	0.5503	−0.1168	0.6984	0.036*	
C14	0.1889 (3)	−0.1398 (2)	0.72083 (8)	0.0291 (4)	
H14	0.0433	−0.1026	0.7031	0.035*	
C15	0.1797 (3)	−0.2443 (2)	0.78105 (9)	0.0318 (4)	
H15	0.3235	−0.2847	0.7992	0.038*	
C16	−0.0463 (3)	−0.2989 (2)	0.82020 (9)	0.0295 (4)	
C17	−0.0310 (3)	−0.4111 (3)	0.88816 (10)	0.0420 (5)	
H17A	0.0712	−0.3535	0.9150	0.050*	
H17B	0.0468	−0.5239	0.8804	0.050*	
C18	−0.2653 (4)	−0.4474 (3)	0.93005 (10)	0.0502 (5)	
H18A	−0.3392	−0.3374	0.9411	0.075*	
H18B	−0.2393	−0.5251	0.9724	0.075*	
H18C	−0.3693	−0.5032	0.9039	0.075*	
O1	1.9060 (2)	0.85667 (18)	0.06800 (7)	0.0537 (4)	
O2	1.7321 (3)	1.10716 (19)	0.02820 (8)	0.0565 (4)	
O3	1.0497 (2)	0.21570 (18)	0.51052 (7)	0.0464 (4)	
O4	−0.2355 (2)	−0.25612 (17)	0.79837 (6)	0.0434 (4)	
H2	1.868 (7)	1.118 (5)	0.001 (2)	0.140 (14)*	

### Atomic displacement parameters (Å^2^)

**Table:**

	*U*^11^	*U*^22^	*U*^33^	*U*^12^	*U*^13^	*U*^23^
C1	0.0422 (10)	0.0350 (9)	0.0294 (9)	−0.0042 (8)	0.0027 (8)	0.0061 (7)
C2	0.0401 (10)	0.0458 (11)	0.0351 (10)	−0.0011 (8)	0.0066 (8)	0.0093 (8)
C3	0.0367 (9)	0.0362 (9)	0.0293 (9)	−0.0033 (7)	0.0052 (7)	0.0037 (7)
C4	0.0362 (10)	0.0399 (10)	0.0306 (9)	−0.0047 (8)	0.0049 (7)	0.0050 (8)
C5	0.0352 (9)	0.0362 (9)	0.0299 (9)	−0.0051 (7)	0.0042 (7)	0.0021 (7)
C6	0.0346 (9)	0.0356 (9)	0.0293 (9)	−0.0053 (7)	0.0039 (7)	0.0041 (7)
C7	0.0310 (9)	0.0343 (9)	0.0308 (9)	−0.0053 (7)	0.0030 (7)	0.0031 (7)
C8	0.0296 (9)	0.0340 (9)	0.0267 (9)	−0.0050 (7)	−0.0010 (7)	0.0041 (7)
C9	0.0275 (8)	0.0321 (9)	0.0274 (8)	−0.0046 (7)	−0.0020 (7)	0.0009 (7)
C10	0.0265 (8)	0.0342 (9)	0.0277 (9)	−0.0034 (7)	−0.0034 (7)	0.0023 (7)
C11	0.0253 (8)	0.0324 (9)	0.0303 (9)	−0.0029 (7)	−0.0032 (7)	0.0015 (7)
C12	0.0275 (8)	0.0322 (9)	0.0278 (9)	−0.0026 (7)	−0.0025 (7)	0.0027 (7)
C13	0.0259 (8)	0.0328 (9)	0.0299 (9)	−0.0028 (7)	−0.0028 (7)	0.0030 (7)
C14	0.0260 (8)	0.0311 (8)	0.0285 (9)	−0.0011 (7)	−0.0032 (7)	0.0017 (7)
C15	0.0253 (8)	0.0345 (9)	0.0324 (9)	−0.0019 (7)	−0.0035 (7)	0.0059 (7)
C16	0.0276 (8)	0.0291 (8)	0.0293 (9)	−0.0010 (7)	−0.0030 (7)	0.0034 (7)
C17	0.0349 (9)	0.0471 (11)	0.0369 (10)	−0.0026 (8)	−0.0018 (8)	0.0157 (8)
C18	0.0468 (12)	0.0550 (12)	0.0389 (11)	−0.0009 (9)	0.0072 (9)	0.0176 (9)
O1	0.0502 (8)	0.0507 (8)	0.0466 (9)	0.0085 (7)	0.0175 (6)	0.0193 (7)
O2	0.0603 (10)	0.0444 (8)	0.0501 (9)	0.0071 (7)	0.0212 (7)	0.0200 (7)
O3	0.0292 (7)	0.0591 (9)	0.0442 (8)	−0.0057 (6)	−0.0048 (6)	0.0166 (6)
O4	0.0275 (6)	0.0587 (9)	0.0382 (7)	−0.0020 (6)	−0.0040 (5)	0.0127 (6)

### Geometric parameters (Å, º)

**Table:**

C1—O1	1.211 (2)	C9—O3	1.215 (2)
C1—O2	1.320 (2)	C9—C10	1.481 (2)
C1—C2	1.497 (3)	C10—C11	1.340 (2)
C2—C3	1.515 (2)	C10—H10	0.9500
C2—H2A	0.9900	C11—C12	1.444 (2)
C2—H2B	0.9900	C11—H11	0.9500
C3—C4	1.522 (2)	C12—C13	1.349 (2)
C3—H3A	0.9900	C12—H12	0.9500
C3—H3B	0.9900	C13—C14	1.440 (2)
C4—C5	1.519 (2)	C13—H13	0.9500
C4—H4A	0.9900	C14—C15	1.339 (2)
C4—H4B	0.9900	C14—H14	0.9500
C5—C6	1.524 (2)	C15—C16	1.476 (2)
C5—H5A	0.9900	C15—H15	0.9500
C5—H5B	0.9900	C16—O4	1.2161 (19)
C6—C7	1.522 (2)	C16—C17	1.502 (2)
C6—H6A	0.9900	C17—C18	1.511 (3)
C6—H6B	0.9900	C17—H17A	0.9900
C7—C8	1.523 (2)	C17—H17B	0.9900
C7—H7A	0.9900	C18—H18A	0.9800
C7—H7B	0.9900	C18—H18B	0.9800
C8—C9	1.508 (2)	C18—H18C	0.9800
C8—H8A	0.9900	O2—H2	0.90 (4)
C8—H8B	0.9900		
			
O1—C1—O2	122.25 (17)	C7—C8—H8A	108.8
O1—C1—C2	124.48 (16)	C9—C8—H8B	108.8
O2—C1—C2	113.26 (16)	C7—C8—H8B	108.8
C1—C2—C3	115.59 (15)	H8A—C8—H8B	107.7
C1—C2—H2A	108.4	O3—C9—C10	121.41 (15)
C3—C2—H2A	108.4	O3—C9—C8	121.50 (15)
C1—C2—H2B	108.4	C10—C9—C8	117.06 (14)
C3—C2—H2B	108.4	C11—C10—C9	121.25 (15)
H2A—C2—H2B	107.4	C11—C10—H10	119.4
C2—C3—C4	111.17 (15)	C9—C10—H10	119.4
C2—C3—H3A	109.4	C10—C11—C12	124.38 (15)
C4—C3—H3A	109.4	C10—C11—H11	117.8
C2—C3—H3B	109.4	C12—C11—H11	117.8
C4—C3—H3B	109.4	C13—C12—C11	122.93 (15)
H3A—C3—H3B	108.0	C13—C12—H12	118.5
C5—C4—C3	113.63 (15)	C11—C12—H12	118.5
C5—C4—H4A	108.8	C12—C13—C14	123.43 (15)
C3—C4—H4A	108.8	C12—C13—H13	118.3
C5—C4—H4B	108.8	C14—C13—H13	118.3
C3—C4—H4B	108.8	C15—C14—C13	124.62 (15)
H4A—C4—H4B	107.7	C15—C14—H14	117.7
C4—C5—C6	112.83 (15)	C13—C14—H14	117.7
C4—C5—H5A	109.0	C14—C15—C16	122.26 (15)
C6—C5—H5A	109.0	C14—C15—H15	118.9
C4—C5—H5B	109.0	C16—C15—H15	118.9
C6—C5—H5B	109.0	O4—C16—C15	121.69 (15)
H5A—C5—H5B	107.8	O4—C16—C17	121.60 (15)
C7—C6—C5	112.67 (15)	C15—C16—C17	116.71 (14)
C7—C6—H6A	109.1	C16—C17—C18	115.05 (15)
C5—C6—H6A	109.1	C16—C17—H17A	108.5
C7—C6—H6B	109.1	C18—C17—H17A	108.5
C5—C6—H6B	109.1	C16—C17—H17B	108.5
H6A—C6—H6B	107.8	C18—C17—H17B	108.5
C6—C7—C8	113.09 (14)	H17A—C17—H17B	107.5
C6—C7—H7A	109.0	C17—C18—H18A	109.5
C8—C7—H7A	109.0	C17—C18—H18B	109.5
C6—C7—H7B	109.0	H18A—C18—H18B	109.5
C8—C7—H7B	109.0	C17—C18—H18C	109.5
H7A—C7—H7B	107.8	H18A—C18—H18C	109.5
C9—C8—C7	113.98 (14)	H18B—C18—H18C	109.5
C9—C8—H8A	108.8	C1—O2—H2	109 (2)
			
O1—C1—C2—C3	12.6 (3)	C8—C9—C10—C11	−176.26 (15)
O2—C1—C2—C3	−168.51 (16)	C9—C10—C11—C12	178.37 (15)
C1—C2—C3—C4	−173.08 (16)	C10—C11—C12—C13	−177.39 (16)
C2—C3—C4—C5	−179.00 (15)	C11—C12—C13—C14	177.91 (15)
C3—C4—C5—C6	−176.13 (15)	C12—C13—C14—C15	−179.56 (16)
C4—C5—C6—C7	−179.98 (14)	C13—C14—C15—C16	178.72 (15)
C5—C6—C7—C8	−178.05 (14)	C14—C15—C16—O4	2.6 (3)
C6—C7—C8—C9	−175.62 (14)	C14—C15—C16—C17	−177.86 (16)
C7—C8—C9—O3	10.3 (3)	O4—C16—C17—C18	−7.0 (3)
C7—C8—C9—C10	−171.76 (14)	C15—C16—C17—C18	173.45 (16)
O3—C9—C10—C11	1.7 (3)		

### Hydrogen-bond geometry (Å, º)

**Table:**

*D*—H···*A*	*D*—H	H···*A*	*D*···*A*	*D*—H···*A*
O2—H2···O1^i^	0.90 (4)	1.78 (4)	2.658 (2)	167.0 (4)

Symmetry code: (i) −*x*+4, −*y*+2, −*z*.
